# Heavy metal levels in milk and fermented milk products produced in the Almaty region, Kazakhstan

**DOI:** 10.14202/vetworld.2020.609-613

**Published:** 2020-04-02

**Authors:** N. B. Sarsembayeva, T. B. Abdigaliyeva, Z. A. Utepova, A. N. Biltebay, S. Zh. Zhumagulova

**Affiliations:** 1 Department of Veterinary and Sanitary Examination and Hygiene, Kazakh National Agrarian University, Almaty, Kazakhstan; 2 Department of Food Biotechnology, Almaty Technological University, Almaty, Kazakhstan

**Keywords:** food products, heavy metals, milk, monitoring, quality, safety

## Abstract

**Background and Aim:**

Monitoring food quality and safety remain a pressing issue. The intermediate results of the study on the residual amounts of heavy and toxic elements in food products are presented herein. The aim of this study was the determination of heavy metals in milk and fermented milk products from basic farms in the Almaty region, Kazakhstan.

**Materials and Methods:**

A total of 120 raw milk samples and 80 cottage cheese samples were analyzed. The milk and cottage cheese samples were subjected to mineralization during the analysis to remove organic impurities and determine the heavy metal concentrations using atomic absorption spectrometry.

**Results:**

The contents of cadmium, lead, and arsenic in all raw materials and food products did not exceed the maximum permissible concentrations.

**Conclusion:**

The dairy samples showed low contents of cadmium, mercury, lead, and arsenic that did not exceed the allowable concentrations for basic farms in the Almaty region.

## Introduction

High-quality and safe food products are essential prerequisites for maintaining food independence in Kazakhstan and represent important tasks of the state policy for healthy nutrition. Humans must consume elements necessary for the body; however, along with this ingestion, a large amount of potentially dangerous chemicals can be consumed [1]. The level of contaminants in raw food materials has increased by a factor of almost five over the past 5 years. Toxic elements can be found in 90% of commonly studied food products. Therefore, breadth and depth of understanding the possible contamination routes of foods and processing techniques that can reduce the harmful effects of food contaminants must be enhanced [2]. Heavy metals represent a special group of food contaminants and the influx of heavy metals into the environment has significantly increased recently due to rapid industrialization and the use of new technologies that require heavy metals. The metals can enter raw milk and dairy products through the food chain [3,4]. The likelihood of negative effects of contaminated food products on the health status of the population increases with an unbalanced diet and insufficient intake of essential food components (i.e., the essential amino acids, proteins, vitamins, and microelements) [5]. Toxic elements are the most significant product contaminants [6]. According to most researchers, toxic heavy metals include Pb, Cu, Zn, Ni, Cd, Co, Sb, Sn, Bi, and Hg, which behave as toxicants and ecotoxicants on excessive exposure to environment. Toxicants include elements and compounds that negatively affect an individual organism or group of organisms, while the ecotoxicants are elements or compounds that are harmful to individual organisms as well as the entire ecosystem. Environmentalists have identified priority toxic metals, including cadmium, copper, arsenic, nickel, mercury, lead, zinc, and chromium, as the most dangerous to human and animal health. Among these metals, mercury, lead, and cadmium are the most toxic. Cadmium and lead are also significant environmental pollutants and are toxic to humans and animals [7]. Cd and Pb are non-biodegradable and their accumulation in the environment is dangerous to agriculture and public health [8].

The issue of monitoring the quality and safety of food products remains an important concern [9-11]. Atomic absorption spectrometry and stripping voltammetry are widely used in the field of analytical chemistry to determine a wide range of elements in food products and raw materials [12,13]. Milk is a wholesome food and is a good source of protein, fat, and essential minerals. Moreover, milk and dairy products represent the main constituents of the diet, especially for infants, schoolchildren, and the elderly. Milk and dairy products are the most diverse natural food products in composition because they contain more than 20 trace elements. Most of these trace elements are essential and essential for human health [14]. Therefore, the determination of heavy metals in milk and dairy products is important for trace elements and nutrition research.

The aim of this study was the determination of heavy metals in milk and fermented milk products from basic farms in the Almaty region, Kazakhstan.

## Materials and Methods

### Ethical approval

Ethical permission was obtained from the ethics committee of the Kazakh National Agrarian University. The samples were collected in accordance with the updated regulatory documents in Enbekshikazakh and Zhambyl districts of the Almaty region, Kazakhstan.

### Materials

The authors obtained data regarding the content of residual amounts of heavy metals (cadmium, lead, arsenic, and mercury) in samples of milk and dairy products obtained from farms in the Almaty region in the period of 2018-2019. The heavy metal contents in raw milk and cottage cheese were analyzed. A total of 120 raw milk samples and 80 cottage cheese samples were analyzed. Each sample was packaged in a plastic bag and frozen at −20°C before analysis. The milk and cottage cheese samples were analyzed for heavy metal content at the Kazakhstan-Japan Innovation Center of the Kazakh National Agrarian University.

### Methods

Food sample preparation was performed using the dry and acid mineralization technique. A nitric acid solution at a rate of 1 cm^3^ per 50 g of the product was added to a sample of the test products, mixed, and placed on an electric stove. Carbonization was carefully performed until the smoke ceased, then a cup was placed in an electric furnace previously adjusted to a temperature of approximately 250°C. Simultaneously, the bowl with the product sample was heated using an infrared lamp. Subsequently, ethyl alcohol was added to the bowl at a rate of 5 cm/1 g of dry matter, closed with a watch glass, and maintained for 24-48 h. Carbonization was performed and after it was completed, the samples were gradually mineralized in an electric furnace where the temperature was raised to 450°C at a rate of 50°C every 30 min. Mineralization was performed at this temperature until gray ash was obtained, and the ash bowl was then removed from the electric furnace after 10-15 h of ashing and cooled to room temperature (21°C). The contents were wetted dropwise using a minimal amount of nitric acid solution. The acid was evaporated to dryness in a water bath and exposed in an oven at temperatures up to 140°C. After cooling, the hinged cup was placed in a cooled electric furnace, and the temperature was gradually raised to 300°C and maintained for 0.5 h. This cycle was repeated several times [15].

To measure the mass concentration of mercury in the product samples, the inversion voltammetry method was performed after preliminary sample preparation with ozonation (1.5-2.5 min). Sample ozonation was performed directly in the analyzer cells using a «ЧиcTo-TA» ozonation device. The mass concentration of mercury in the test sample solutions was determined by adding certified mixtures with established mercury contents [16].

The mass concentrations of arsenic in the samples were determined by adding a certified As(III) mixture to the analyzed solution. The total arsenic concentration was determined after the reduction of As(V) compounds to As(III) sodium using sodium pyrosulfite or hydrazine sulfate on evaporation of the sample in the presence of sulfuric acid. To determine the As(V) content in milk, the sample was evaporated to dryness on the addition of concentrated hydrochloric acid. The dry residue was placed in a muffle at >450°C. The As(V) remaining in the sample was reduced to As(III) with sodium pyrosulfite, after which As(III) was determined in mineralizate. The mass concentration of As(III) was determined by taking the difference between the total concentration of arsenic and mass concentration of As(V) [17].

To determine the concentration of lead and cadmium, the ash was dissolved in a crucible while being heated in nitric acid (1:1; 1-5 cm^3^ of acid per sample, depending on the product ash content). The solution was evaporated to wet salts and the precipitate was dissolved in 15-20 cm^3^ of nitric acid with a mass fraction of 1%. The sample was then quantitatively transferred to a volumetric flask with a capacity of 25 cm^3^ and adjusted to the mark using the same acid. The spectrophotometer was subsequently prepared for operation, and the measurement conditions were selected [18].

The studies were performed while considering repeatability and intermediate precision. When calculating the elemental concentrations in the samples, the metrological processing of the results was performed in accordance with the relevant regulations [19].

The concentration of toxic heavy metals in the milk samples and dairy products was determined using a novAA 350 atomic absorption spectrometer (Analytik Jena, Germany) using a TaLab voltammetric analyzer. This is a next-generation instrument for automated analysis using flame atomic absorption spectroscopy with a deuterium correction of background radiation (deuterium lamp with a hollow cathode) with the ability to quickly switch to determination modes by atomic emission spectroscopy without using a hollow cathode lamp.

## Results and Discussion

The results of the analysis of toxic heavy metals in cow milk from basic farms in the Almaty region are listed in Table-1.

**Table-1:** Data regarding the content of toxic heavy metals in cow milk from basic farms in the Almaty region.

Tested samples	*Content of heavy and toxic metals, ppm*							

	*Cd*	MAC	*Pb*	MAC	*As*	MAC	*Hg*	MAC
K/1-4-1-1	0.0029	0.03	0.0010	0.1	n/a	0.05	n/a	0.005
K/1-4-1-2	0.0025	0.03	0.008	0.1	n/a	0.05	n/a	0.005
K/1-4-1-3	0.0029	0.03	0.0010	0.1	n/a	0.05	n/a	0.005
A/1-4-1-1	0.0027	0.03	0.0010	0.1	n/a	0.05	n/a	0.005
A/1-4-1-2	0.0027	0.03	0.0010	0.1	n/a	0.05	n/a	0.005
A/1-4-1-3	0.0027	0.03	0.0010	0.1	n/a	0.05	n/a	0.005

K=K farm, A=A farm

Cadmium is the most dangerous of heavy metals, with pronounced carcinogenic and mutagenic properties [20,21]. From Table-1, the cadmium content in the milk samples varied from 0.0025 to 0.0029 ppm, which did not exceed the maximum allowable concentration (MAC).

Lead is classified as a poison that primarily affects human nervous and vascular systems. The mechanism of the toxic action of lead can be explained by its ability to block sulfhydryl groups in enzymes involved in porphyrin synthesis [22]. The lead content in the tested milk samples ranged from 0.0010 to 0.008 ppm, with an average of 0.0045 ppm.

The concentrations of mercury, arsenic, cadmium, and lead in cottage cheese were also determined. The results of the heavy metal analyses in the cottage cheese samples from the K and A farms are listed in Table-2. From Table-2, the cadmium content in the cottage cheese samples ranged from 0.0446 to 0.0570 mg/g, while the lead content ranged from 0.0089 to 0.0162 mg/g, which did not exceed the MAC.

**Table-2:** Data regarding the content of toxic heavy metals in cottage cheese from the basic farms in the Almaty region.

Tested samples	*Content of heavy and toxic metals, mg/kg*							

	*Cd*	MAC (mg/kg)	*Pb*	MAC (mg/kg)	*As*	MAC (mg/kg)	*Hg*	MAC (mg/kg)
K/1-5-1-1	0.0496	0.1	0.0089	0.3	n/a	0.2	n/a	0.01
K/1-5-1-2	0.0566	0.1	0.0104	0.3	n/a	0.2	n/a	0.01
K/1-5-1-3	0.0498	0.1	0.0093	0.3	n/a	0.2	n/a	0.01
A/1-5-1-1	0.0546	0.1	0.0101	0.3	n/a	0.2	n/a	0.01
A/1-5-1-2	0.0570	0.1	0.0132	0.3	n/a	0.2	n/a	0.01
A/1-5-1-3	0.0446	0.1	0.0117	0.3	n/a	0.2	n/a	0.01
A/1-5-1-4	0.0543	0.1	0.0138	0.3	n/a	0.2	n/a	0.01
A/1-5-1-5	0.0486	0.1	0.0162	0.3	n/a	0.2	n/a	0.01

K=K farm, A=A farm

The arsenic and mercury contents in these products were lower than both the established standards and detection limit of the device. Therefore, it is currently impossible to assess the fluctuations in the contents of these trace elements.

Despite the results described above, the possibility that milk and dairy products exceeding the standards for these indicators (MACs) cannot be excluded. Since environmental pollution due to these metals increases every year, this can lead to the appearance of these contaminants in the dairy industry at some point.

**Figure-1:**
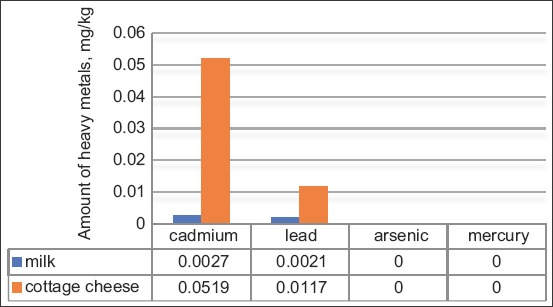
Comparative data regarding the heavy metal contents in the milk and cottage cheese samples.

The analysis of some toxic substances in the milk and dairy products revealed that the average concentration of heavy metals did not exceed the respective MACs. Comparative data of the heavy metal contents in milk and cottage cheese are listed in Figure-1.

It should be noted that the cadmium, lead, mercury, and arsenic contents in all studied samples of raw materials (milk) and food products (cottage cheese) did not exceed the respective MACs. Thus, it can be concluded that the oral intake of these heavy metals from the tested food products is quite low.

Atomic absorption spectrometry and stripping voltammetry provide rapid analyses of toxic element contents, which allow for the control of contaminated products in markets and can potentially dangerous and low-quality food to be sold and negatively affect human health [23].

Data regarding the content of residual heavy metals were also obtained. The arsenic and mercury contents of cottage cheese were below the established standards. According to the analysis results, the content of lead and cadmium in the cottage cheese made from milk from farm A did not exceed the threshold limit value (TLV). The average cadmium concentration was 0.0520±0.03 mg/kg, while for the lead, the average concentration was 0.0116±0.01 mg/kg. The heavy metal contents in the cottage cheese from farm K also did not exceed the TLV with average values of 0.052 and 0.0095 mg/kg for cadmium and lead, respectively. Of all samples tested for environmental safety, the maximum amount of heavy metals (Cd and Pb) was found in the cottage cheese samples in the following order: Cd>Pb>As>Hg.

Lante *et al*. [24] reported that the concentration of toxic heavy metals in milk and dairy products depends largely on the farm location. Therefore, several studies have been conducted to evaluate the metal content in milk from various regions. The residual amounts of heavy metals in this study are likely associated with contamination of animal feed and water, which can manifest in the produced milk and dairy products at various levels [25] and can be transferred to products through processing procedures. Similar results were reported by Tripathi [26]. It should be noted that the lead content was lower than the regulated limit of 1 mg/kg established in relevant food regulations [27]. According to the results reported by Gabryszuk *et al*. [28], the concentration of Pb in cow milk from organic farms was significantly higher, ranging from 0.0041 to 0.0062 μg/ml. The cadmium concentration in cottage cheese from A farm was higher than that in the raw material of the K farm. The average cadmium concentration in all samples approached the maximum permissible level (0.0519). The average heavy metal concentrations in the samples were comparable with the data reported by the International Dairy Federation [29].

However, the cadmium and lead concentration increased 1.5-2.0 times in the cottage cheese obtained from the K farm milk compared to the raw materials obtained from the A farm milk. This can be explained by the selective binding of pollutants to proteins, and cottage cheese contains proportionally higher protein content.

## Conclusion

Cow milk and dairy products from the A and K farms showed a favorable mineral composition and low concentrations of toxic heavy metals. The dairy samples showed low contents of cadmium, mercury, lead, and arsenic that did not exceed the allowable concentrations for basic farms in the Almaty region.

A comparative analysis of the toxic element content in feed and their MACs revealed opportunities to obtain environmental-friendly livestock products from basic farms in the Almaty region. Previously established conversion factors for heavy metals in the soil-feed-milk-food chain can be used to develop environmentally sound milk production technologies.

The interim results obtained in 2018-2019 and their reliability were confirmed by a collection of milk and cottage cheese samples, as well as by conducting relevant experiments using modern research methods. The reliability and traceability of the reported results are confirmed by the previous studies and other related documentation. The activities of the project executors were regulated by the provisions approved by the Local Ethics Commission at Kazakh National Agrarian University.

## Authors’ Contributions

NBS: Structured the experimental design, organized, and conducted statistical analyses. TBA: Data collection, literature review, and manuscript preparation. ZAU: Formulated the problem and hypothesis. ANB: Results interpretation. SZZ: Data collection. All authors read and approved the final manuscript.
